# Assessing reliability and validity of the Chinese version of the Crown-Crisp Experience Index and its application in pneumoconiosis patients

**DOI:** 10.1186/s12888-023-04731-x

**Published:** 2023-04-18

**Authors:** Fulin Cai, Sheng Xue, Mei Zhang, Xiufeng Chen, Jing Zhang, Yi Bao, Yaqiang Li

**Affiliations:** 1 The First Affiliated Hospital of Anhui, University of Science and Technology, Huainan, China; 2 Anhui University of Science and Technology, Huainan, China; 3 Shool of Mental Health, Bengbu Medical College, Bengbu, China; 4 Prevention and Treatment Hospital for Occupational Diseases, Huainan, China

**Keywords:** Pneumoconiosis, Anxiety, Phobia, Factor analysis

## Abstract

In China, among all patients with occupational diseases, 90% have pneumoconiosis. The disease, which leads to psychological problems, seriously affects patients’ lives. The Crown-Crisp Experience Index (CCEI) is a multidimensional questionnaire to assess patients’ psychological conditions. Yet there is no Chinese version of CCEI. This study, therefore, aims to develop a Chinese CCEI, in line with standard localization procedures, by translating, back-translating, and culturally adapting the original English version. The final Chinese version comprises 47 items in six dimensions. The reliability and validity of the Chinese CCEI were tested by analyzing the data collected from 1,000 pneumoconiosis patients from an occupational disease prevention and treatment hospital. A rank sum test was carried out to compare the phobic anxiety (PHO) between pneumoconiosis patients and retired miners. The results of exploratory factor analysis show six principal components, which explain a total of 78.246% variances. Confirmatory factor analysis shows that the Chi-square freedom ratio (*χ*^*2*^*/df*) were less than 3, the root mean square error approximation (RMSEA) were less than 0.05, comparative fit Index(CFI) and incremental fit index (IFI) were greater than 0.9, average variance extracted(AVE) in six dimensions were less than 0.5, residual variances(CR) were greater than 0.8, Cronbach’s alpha coefficient 0.839, Omega ω coefficient 0.889, and S-CVI 0.88. The PHO of pneumoconiosis patients was significantly higher than that of retired miners exemplified by a statistical difference (*P* < *0.05*). The study shows that the Chinese version of CCEI enjoys a high degree of reliability and validity and thus can be used as a screening tool for measuring patients’ anxiety and fear levels.

## Introduction

Coal Workers’ Pneumoconiosis (CWP), biologically characterized by diffuse fibrosis of lung tissues, is a systemic disease mainly caused by long-term inhalation of mineral dust during occupational activities such as coal mining and cement planting. The symptoms of pneumoconiosis include cough, sputum expectoration, chest pain, and dyspnea. The disease affects not only the respiratory system but digestive and nervous systems as well. In severe cases, it can be life-threatening. In 2018, the incidence of occupational diseases in China exceeded 975,000, of which 90% were pneumoconiosis [1]. Pneumoconiosis is a chronic disease that cannot be cured 100% except through lung transplantation [2]. With pulmonary fibrosis, pneumoconiosis patients suffer from weakening lung function and decreased immunity [3]; their health is easily threatened by respiratory infections. Burdened by long-term pain and the disease’s chronic traits, patients are likely to develop anxiety, fear, pessimism, depression, and other negative mindsets [4]. It’s devastating to patients’ lives because the physical and psychological infliction leads to low treatment compliance and worrying treatment efficiency. A timely recognition of patients’ psychological status and corresponding interventions, therefore, is an elixir to improve their compliance and well-being.

To evaluate patients’ psychological status, most Chinese researchers use internationally recognized tools including the Self-rating Anxiety Scale (SAS) [5], Self-rating Depression Scale (SDS) [6], and Depression Anxiety Stress Scale-21 (DASS-21) [7]. These tools focus more on a certain aspect of psychological problems, whereas Somatic Symptom Scale SCL-90 [8], though being more comprehensive, has a large number of items, and the long-term survey process may make respondents impatient. This is especially true in China, where outpatient service is heavily burdened because of limited medical resources [9]. Therefore, there is an urgent need for a measurement tool that can assess aspects of psychology in a short period of time to help medical workers quickly screen for problems in one or more aspects.

Crown-Crisp Experience Index (CCEI) is a quantitative tool that evaluates patients’ phobia index through a questionnaire. Compiled in 1966 by Crown and Crisp, professors of psychiatry at Middlesex Hospital in London, UK, the questionnaire covers six dimensions with eight items each. Specifically, the six dimensions are free-floating anxiety (FFA), phobic anxiety (PHO), obsessive–compulsive traits and symptoms (OBS), somatic symptoms (SOM), depressive symptoms (DEP), and hysterical traits and symptoms (HYS) [10]. It is a succinct self-rating scale for common phobias and anxiety disorders. CCEI can be used, for instance, to evaluate claustrophobia, pathophobia, acrophobia, and demophobia levels. Each item ranges from 0 to 2 points and each dimension 0–16. The total score of the questionnaire falls into 0–96. Notably, the survey has “reversed items,” meaning the higher the score, the greater the degree of anxiety and phobia. The questionnaire was widely used in clinical research on heart disease [11], tinnitus [12], anorexia nervosa [13], ovarian cancer [14], and even children’s attention deficit hyperactivity disorder (ADHD) [15]. To date, there is no Chinese version of CCEI. This study thus aims to introduce the Chinese CCEI questionnaire and test its reliability and validity by use of EFA, CFA, Cronbach’s α coefficient, CVI, and so on to provide an effective tool to measure anxiety and fear of CWP patients.

Exploratory Factor Analysis (EFA) and Confirmatory Factor Analysis (CFA) are recognized as reliable methods for testing the stability and structural rationality of questionnaires and are widely used [16]. EFA is a method to test the relationship between observed and latent variables through factor loading. Before performing data analysis, Kaiser-Meyer-Olkin is used to determine sampling adequacy. If the result is greater than 0.90, then the sample can be used for factor analysis [17]. CFA tests the established model structural assumptions by assessing the goodness of fit [18]. The reliability of the original CCEI questionnaire was tested by reliability coefficients (Split-half: FFA = 0.85, PHO = 0.73, DEP = 0.65, OBS = 0.43, SOM = 0.37 HYS = 0.63) [10]. In 1998, the Kappa coefficient of the total questionnaire on 248 samples was 0.88 [19]. To ensure the reliability of the Chinese version of the CCEI, this study uses a cultural adaptation method and applies the final Chinese version to patients with pneumoconiosis. Using factor analysis to test the reliability and validity of the questionnaire is necessary and suitable.

## Materials and methods

### The CCEI questionnaire translation and cross-cultural adaptation

**Fig. 1:**
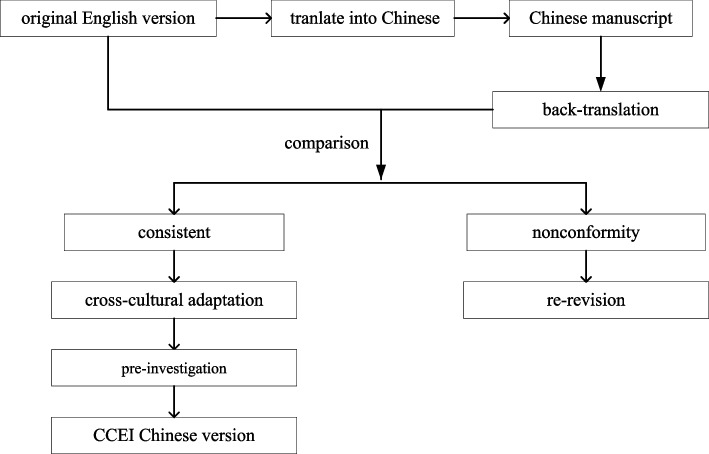
The localization process of the Chinese CCEI

Following the principle of cross-cultural adaptation [20], the process of localization of Chinese CCEI is shown in Fig. 1. Following are the procedures: (1) Translation: One English major and one psychiatry major were invited to translate the original English CCEI into Chinese, and then two experts in psychiatry integrated the two translations into the first draft after deliberation and discussion. (2) Back translation: Two medical majors with English language-speaking backgrounds re-translated the first draft of the Chinese CCEI into English. The author didn’t provide the original copy to translators to avoid affecting translation results. The re-translated copy was then compared with the original to see if the expression was consistent. (3) Cross-cultural adaptation: 15 experts (Table 1) in medical-related fields evaluated the expression and made it more idiomatic. Relevant forms were filled in by invitees, and the first round of expert consultation was carried out. Controversial items underwent a second round of expert consultation. After two rounds (response rate 100%), expert judgment coefficient (Ca), which indicates the determination level of experts, was 0.93, and familiarity degree coefficient (Cs), was 0.89. According to the formula authority coefficient (Cr) = (Ca + Cs)/2 [21], the expert authority coefficient was 0.91 > 0.9 (Table 2). Consequently, 15 experts, taking into account cultural differences, unanimously suggested deleting item 9, i.e. *Do you think that “cleanliness is next to godliness?”*. The questionnaire finally comprised 47 items in six dimensions.

**Table 1:** Biodata of experts

	Number of People	Ratio (%)
Age		
40–49	6	40
≥ 50	9	60
Educational Background		
Master	8	53.3
Ph.D	7	46.7
Field of Work		
Clinical Medicine	4	26.7
Psychiatry	5	33.3
Nursing	3	20
Occupational Safety and Health	3	20

**Table 2:** Experts’ authority coefficient

Number of People	Ca	Cs	Cr
15	0.93	0.89	0.91

### Pre-investigation

To assess the acceptability of the questionnaire, the parameters for pilot study include time taken to fill the questionnaire, the format, clarity of the content, scoring and result interpretation. In this study, 50 questionnaires were distributed to CWP patients using the convenience sampling method from an occupational disease prevention hospital for pilot study. A 3-point scoring method was adopted; that is, answering “yes” tallies 2 points, “sometimes” 1 point, and “no” 0. For reverse items, the scoring is exactly the opposite. For example, Item 5 in the questionnaire: *Can you think as quickly as you used to?* A “yes” answer to this item scores 0, the “sometimes” answer scores 1, and the “no” answer scores 2. Due to low literacy levels and the old age of the subjects, this study conducted one-to-one surveys by six research members who received the same training and paid attention to the subjects’ opinions and suggestions toward each item. The format, content, item options, and filling-out time of the questionnaire were all reportedly reasonable. It takes 12–15 min to complete the questionnaire.

### Participants

To ensure reliability and validity of the questionnaire, the number of interviewees should be 5–10 times the number of questionnaire items [22]. Given the possibility of invalid responses and the need to meet CFA and EFA test standards, 1,000 CWP patients were invited in this study. The participants were patients who meet the following criteria: diagnosed with pneumoconiosis and voluntary. Patients were dismissed from the study if they were discharged or out of the hospital during the survey period or had difficulties communicating due to language, hearing, and intellectual impairments or were diagnosed with other severe diseases. Another 100 healthy retired miners were also recruited as the control group. The retired miners in China undergo regular health check in designated occupational health check centers. The participants were recruited among those who attended the occupational health checks. The participants must meet the following criteria: (1) the miner has to be retired for more than 1 year; (2) the miner has to be pneumoconiosis-free and without malignant tumors; (3) the miner has no hearing and comprehension impairment, and is easy to communicate; (4) the miner must be voluntary. The participants have to register their personal information, attend the occupational health checks and sign voluntary consent prior to undertake the questionnaire survey. A total of 136 participants were invited and only 100 actually participated in the questionnaire survey with a participation rate of 73.5%.

### Data collection

The one-to-one surveys were conducted face-to-face by six research members who received the same training. A total of 1,000 questionnaires were collected in 7 days. Among them, 15 were incomplete and the remaining 985 were valid.They were randomly divided into 2 groups, one group was used to do EFA (*n* = 493) and the other one was for CFA (*n* = 492). The effective rate of the survey was 98.5%, which was in line with the survey standard. For test–retest reliability, this study conducted a second face-to-face survey on the 15th day after the first one, conforming to the test–retest reliability time ranging from 2 weeks to 1 month [23].

### Statistical analysis

Data software Epidata 3.1 was used to input data which were then randomly divided into two groups. One group was used to do EFA (*n* = 493) through SPSS 22.0, and the other was for CFA (*n* = 492) using AMOS 25.0. The reliability and validity of the study were tested by Cronbach’ α, composite reliability, CR ω coefficient, ICC, and goodness of fit indices, which should meet the following criteria (17): χ2/*df* < 3, GFI > 0.9, RMSEA < 0.05, NFI > 0.9, and CFI > 0.9. Content validity index (CVI) and correlation coefficient were used to test reliability and validity of the Chinese CCEI. Quantitative aspects of the study were described by composition ratio, mean, standard deviation and M (SD).

## Results

### General information

The type of work in question happens in specific scenarios, so the survey subjects were all males, age 47–94 with M(SD) 75.35 (10.54) years. The subjects had an average 24.77 (9.68) years of exposure to dust. Diagnosis of pneumoconiosis is common after 20 years of exposure due to the asymptomatic nature of the disease. Most pneumoconiosis patients as a result are elderly and characterized by low educational levels (94.9% of them have junior high school education or below). See Table 3 for details.

**Table 3:** Biodata of pneumoconiosis patients (*n* = 985)

	Number of People	Ratio (%)
Age		
≤ 59	151	15.3
60–69	221	22.4
70–79	350	35.5
≥ 80	263	26.7
Disease Course		
≤ 9	266	27.0
10–19	256	26.0
20–29	249	25.3
≥ 30	214	21.7
Phase of Pneumoconiosis		
One	812	82.4
Two	104	10.6
Three	69	7.0
Educational Background		
Junior high or below	920	93.4
Senior high	36	3.7
College or above	29	2.9

### Data analysis

#### Exploratory factor analysis of CCEI Chinese version

The KMO and Bartlett test result (see Table 4) of the CCEI questionnaire for CWP patients is 0.904, greater than 0.9, allowing the performance of factor analysis. Varimax with Kaiser normalization is then applied to extract 6 factors from 47 items (Eigenvalues over > 1). The six factors are: DEP (items 5, 11, 17, 23, 29, 35, 41, 47), FFA (items 1, 7, 13, 20, 25, 31, 37, 43), PHO (items 2, 8, 14, 19, 26, 32, 38, 44), SOM (items 4, 10, 16, 22, 28, 34, 40, 46), HYS (items 6, 12, 18, 24, 30, 36, 42, 48), and OBS (items 3, 15, 21, 27, 33, 39, 45). According to the study, a total of 78.246% variance is explained. The detailed information is shown in Table 5.

**Table 4:** KMO and Bartlett Test of CCEI (*n* = 493)

Kaiser–Meyer–Olkin statistics with adequate samples		.904
Bartlett test	Approximate Chi-square	18,963.608
	df	1128
	Sig	.000

**Table 5:** Component matrix of each dimension of the CCEI (*n* = 493)

Item		Component					
		1	2	3	4	5	6
1	Do you often feel upset for no obvious reason?		0.512				
7	Have you felt as though you might faint?		0.526				
13	Do you feel uneasy and restless?		0.702				
20	Do you feel uneasy travelling on buses or the Underground even if they are not crowded?		0.621				
25	Would you say you were a worrying person?		0.760				
31	Do you often feel “strung-up” inside?		0.517				
37	Have you ever had the feeling you are “going to pieces”?		0.582				
43	Do you have bad dreams which upset you when you wake up?		0.662				
2	Do you have an unreasonable fear of being in enclosed spaces such as shops, lifts, etc.?			0.645			
8	Do you find yourself worrying about getting some incurable illness?			0.428			
14	Do you feel more relaxed indoors?			0.469			
19	Do you sometimes feel really panicky?			0.507			
26	Do you dislike going out alone?			0.518			
32	Do you worry unduly when relatives are late coming home?			0.685			
38	Are you scared of heights?			0.521			
44	Do you feel panicky in crowds?			0.662			
3	Do people ever say you are too conscientious?						0.628
15	Do you find that silly or unreasonable thoughts keep recurring in your mind?						0.538
21	Are you happiest when you are working?						0.628
27	Are you a perfectionist?						0.558
33	Do you have to check things you do to an unnecessary extent?						0.516
39	Does it irritate you if your normal routine is disturbed?						0.607
45	Do you find yourself worrying unreasonably about things that do not really matter?						0.533
4	Are you troubled by dizziness or shortness of breath?				0.457		
10	Do you often feel sick or have indigestion?				0.547		
16	Do you sometimes feel tingling or pricking sensations in your body, arms or legs?				0.549		
22	Has your appetite got less recently?				0.491		
28	Do you feel unduly tired and exhausted?				0.636		
34	Can you get off to sleep alright at the moment?				0.683		
40	Do you often suffer from excessive sweating or fluttering of the heart?				0.525		
46	Has your sexual interest altered?				0.602		
5	Can you think as quickly as you used to?	0.725					
11	Do you feel that life is too much effort?	0.611					
17	Do you regret much of your past behaviour?	0.598					
23	Do you wake unusually early in the morning?	0.681					
29	Do you experience long periods of sadness?	0.702					
35	Do you have to make a special effort to face up to a crisis or difficulty?	0.665					
41	Do you find yourself needing to cry?	0.785					
47	Have you lost your ability to feel sympathy for other people?	0.771					
6	Are your opinions easily influenced?					0.728	
12	Have you, at any time in your life, enjoyed acting?					0.735	
18	Are you normally an excessively emotional person?					0.641	
24	Do you enjoy being the centre of attention?					0.639	
30	Do you find that you take advantage of circumstances for your own ends?					0.712	
36	Do you often spend a lot of money on clothes?					0.644	
42	Do you enjoy dramatic situations?					0.622	
48	Do you sometimes find yourself posing or pretending?					0.569	
Eigenvalue		6.932	6.521	6.491	6.425	6.295	5.722
Total variance explained (%)		15.841	13.665	13.225	13.086	12.021	10.408

Extraction method: principal components; Rotation method: varimax with Kaiser normalization

#### Confirmatory factor analysis of CCEI Chinese version

**Fig. 2:**
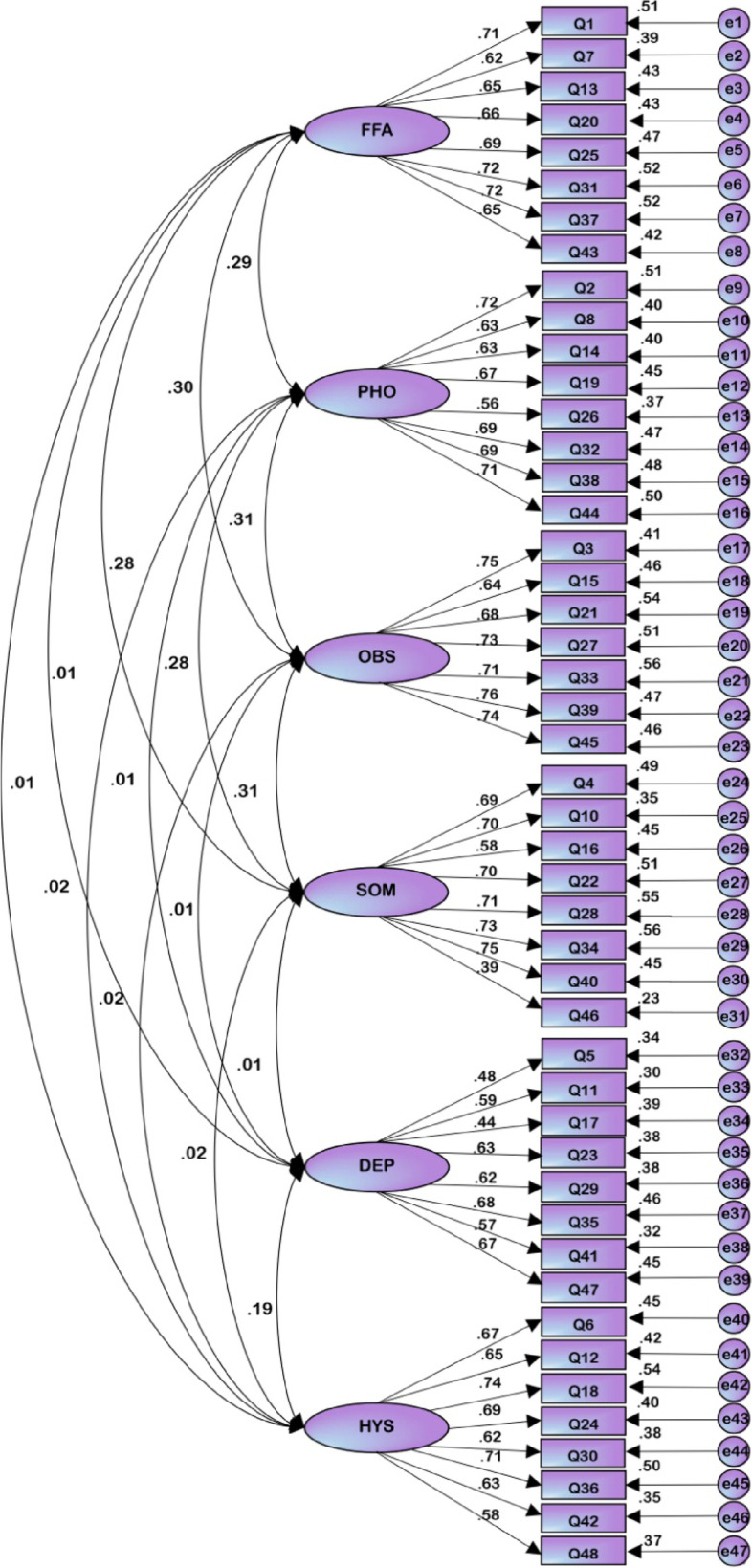
Path diagram of each dimension

Structural Equation Modeling (SEM) is essentially a path analysis model with latent variables [18]. In this study, 47 items are observation variables, and FFA, PHO, OBS, SOM, DEP, and HYS are latent variables. AMOS 25.0 is used to draw a path diagram to verify the theoretical structure of exploratory factor analysis, as shown in Fig. 2. Confirmatory factor analysis shows that the fit indexes of the structure are acceptable and reasonable, as shown in Table 6. And the CR value of the six dimensions is greater than 0.6, and the AVE value is greater than 0.5, indicating that the items in the CCEI scale have a certain degree of discrimination and can identify the degree of response of different investigators [24], see Table 7.

**Table 6:** Fit indexes of Chinese CCEI (*n* = 492)

Index	χ^2^	*df*	*P*	χ^2^/ *df*	CFI	IFI	RMSEA (90%CI)
CFA	1771.774	930	0.052	1.905	0.912	0.906	0.044 (0.40–0.47)

*df* Degree of freedom, *CFI* Comparative Fit Index, *IFI* Incremental Fit Index, *RMSEA* Root mean square error of approximation, *90% CI* 90% confidence interval for RMSEA

**Table 7:** Parameters estimation result (*n* = 492)

Path Diagram			Unstd	S.E	t-value	std	CR	AVE
Q1	<---	FFA	1.000			0.711		
Q7	<---	FFA	0.862	0.067	12.921	0.622		
Q13	<---	FFA	0.947	0.070	13.581	0.654		
Q20	<---	FFA	0.952	0.070	13.686	0.659		
Q25	<---	FFA	1.007	0.071	14.235	0.685		
Q31	<---	FFA	1.053	0.071	14.934	0.720		
Q37	<---	FFA	1.012	0.068	14.915	0.719		
Q43	<---	FFA	0.921	0.069	13.395	0.645	0.871	0.501
Q2	<---	PHO	1.000			0.716		
Q8	<---	PHO	0.883	0.067	13.101	0.630		
Q14	<---	PHO	0.880	0.067	13.084	0.630		
Q19	<---	PHO	0.934	0.067	13.948	0.671		
Q26	<---	PHO	0.763	0.066	11.622	0.559		
Q32	<---	PHO	0.966	0.068	14.303	0.688		
Q38	<---	PHO	0.958	0.067	14.367	0.692		
Q44	<---	PHO	1.012	0.069	14.703	0.708	0.862	0.506
Q3	<---	OBS	1.000			0.750		
Q15	<---	OBS	0.849	0.062	13.798	0.639		
Q21	<---	OBS	0.920	0.062	14.786	0.681		
Q27	<---	OBS	0.967	0.060	16.030	0.733		
Q33	<---	OBS	0.942	0.061	15.538	0.713		
Q39	<---	OBS	1.017	0.061	16.735	0.763		
Q45	<---	OBS	1.125	0.066	16.462	0.744	0.866	0.510
Q4	<---	SOM	1.000			0.688		
Q10	<---	SOM	0.985	0.070	13.992	0.697		
Q16	<---	SOM	0.822	0.070	11.741	0.578		
Q22	<---	SOM	0.989	0.070	14.088	0.702		
Q28	<---	SOM	1.019	0.071	14.263	0.711		
Q34	<---	SOM	1.050	0.072	14.568	0.728		
Q40	<---	SOM	1.074	0.072	14.923	0.748		
Q46	<---	SOM	0.554	0.069	8.003	0.388	0.840	0.500
Q5	<---	DEP	1.000			0.482		
Q11	<---	DEP	1.229	0.139	8.823	0.585		
Q17	<---	DEP	0.898	0.121	7.432	0.443		
Q23	<---	DEP	1.324	0.145	9.156	0.628		
Q29	<---	DEP	1.289	0.142	9.071	0.617		
Q35	<---	DEP	1.421	0.150	9.501	0.677		
Q41	<---	DEP	1.184	0.137	8.672	0.567		
Q47	<---	DEP	1.421	0.150	9.451	0.670	0.836	0.500
Q6	<---	HYS	1.000			0.672		
Q12	<---	HYS	0.999	0.079	12.585	0.650		
Q18	<---	HYS	1.093	0.078	14.023	0.737		
Q24	<---	HYS	1.014	0.078	13.069	0.678		
Q30	<---	HYS	0.912	0.076	12.038	0.618		
Q36	<---	HYS	1.038	0.077	13.549	0.707		
Q42	<---	HYS	1.018	0.106	10.525	0.629		
Q48	<---	HYS	0.840	0.074	11.325	0.578	0.848	0.500

#### Internal consistency coefficient of CCEI Chinese version

The Cronbach’s αcoefficient of each dimension of this questionnaire and the total questionnaire was 0.839–0.936 > 0.70 and the split-half reliability > 0.7. After 15 days, the survey was repeated. The result showed the ICC of each dimension and the questionnaire ranged from 0.881 to 0.938 and *p* value < 0.05 (Table 8). Composite reliability results showed that the Omega ω coefficient of the 47 items was 0.889, indicating that the questionnaire has good internal consistency [25].

**Table 8:** Reliability result of the CCEI (*n* = 493)

	Item number	Cronbach’s Alpha	Split-half reliability	ICC (*P)*
Chinese CCEI	47	0.885	0.893	0.905 (*P* < 0.001)
FFA	8	0.936	0.905	0.921 (*P* < 0.001)
PHO	8	0.922	0.918	0.902 (*P* < 0.001)
OBS	7	0.906	0.928	0.938 (*P* < 0.001)
SOM	8	0.838	0.880	0.912 (*P* < 0.001)
DEP	8	0.917	0.920	0.908 (*P* < 0.001)
HYS	8	0.908	0.925	0.909 (*P* < 0.001)

#### Pearson correlation analysis of CCEI Chinese version

After Pearson correlation analysis of each dimension of the questionnaire, it can be seen (Table 9) that the correlation coefficient among dimensions is 0.09–0.35, lower than the coefficient between each dimension and the questionnaire, 0.39–0.54. Each dimension has a weak to moderate correlation with each other, whereas each dimension has a pronounced correlation with the questionnaire.

**Table 9:** Correlation analysis between each variable and the questionnaire (*n* = 493)

	PHO	OBS	HYS	SOM	FFA	DEP	Total
PHO	1	0.13^a^	0.24^b^	0.10	0.28^b^	0.19^b^	0.54^b^
OBS		1	0.11	0.21^b^	0.11	0.09	0.40^b^
HYS			1	0.12^a^	0.16^a^	0.21^b^	0.48^b^
SOM				1	0.14^a^	0.17^a^	0.49^b^
FFA					1	0.35^b^	0.42^b^
DEP						1	0.39^b^
Score							1

^a^Significantly correlated at the .05 level (both sides)

^b^Significantly correlated at the .01 level (both sides)

#### Content validity of CCEI Chinese version

CVI is sub-divided into item-level CVI (I-CVI) and scale-level CVI (S-CVI). The 15 experts in this study assessed four aspects of the content validity including semantic equivalence, idiomatic equivalence, experience equivalence, conceptual equivalence, and the results show that I-CVI is 0.73–1.00, S-CVI is 0.88, all greater than 0.7, indicating good content validity [26].

#### Scores comparison between CWP patients and retired miners

The general information of 100 retired miners and 100 patients with pneumoconiosis is shown in Table 10. An independent samples t-test showed that the *p* value was greater than 0.05, and the chi-square test *p* value was greater than 0.05 in terms of educational background, showing no statistical difference. In terms of CCEI score, retired miners scores were between 3 and 44, and M(SD) was 14.56(7.575); CWP patients scores were between 6 and 65, and M(SD) was 19.78(10.404). Table 11 shown that the total score difference between the two group was Z = –3.781, *P* < 0.05, Mann–Whitney U = 3453.500, Wilcoxon W = 8503.500. The PHO of CWP patients was slightly higher than that of retired miners, and *p* value in this dimension was 0.000, which was statistically significant.

**Table 10:** General information of retired miners and CWP patients

Group	Number	Age	Education		Years of exposure to dust
			Junior high or below	Senior high or above	
Retired Miners	100	68.21(8.82)	88	12	26.41 (9.20)
CWP patients	100	70.45(9.52)	94	6	25.39 (8.76)
Result		1.82^a^	2.198^b^		1.42^a^
*P*		0.061	0.138		0.064

^a^ is t value; ^b^ is χ^2^ value

**Table 11:** Score comparison between retired miners and CWP patients

	Component	M (SD)	Cl95%	Mean Rank	Z	*P*
CCEI	Retired Miners	14.56 (7.575)	13.06–16.06	85.04	−3.781	0.000
	CWP Patients	19.78 (10.404)	17.72–21.84	115.97		
FFA	Retired Miners	2.35 (4.145)	1.53–3.17	97.01	−0.949	0.342
	CWP Patients	2.79 (4.176)	1.96–3.62	104.00		
PHO	Retired Miners	3.41 (3.147)	2.79–4.03	75.35	−6.171	0.000
	CWP Patients	7.16 (4.371)	6.29–8.03	125.64		
OBS	Retired Miners	1.74 (3.209)	1.10–2.38	100.48	−0.006	0.996
	CWP Patients	1.88 (3.444)	1.20–2.56	100.52		
SOM	Retired Miners	2.70 (4.756)	1.76–3.64	98.53	−0.535	0.593
	CWP Patients	3.14 (4.985)	2.15–4.13	102.48		
DEP	Retired Miners	2.23 (4.385)	1.36–3.10	96.24	−1.190	0.234
	CWP Patients	3.14 (4.909)	2.17–4.11	104.77		
HYS	Retired Miners	1.86 (4.259)	1.01–2.71	95.61	−1.468	0.142
	CWP Patients	2.37 (4.550)	1.47–3.27	105.40		

## Discussions

### Reliability and validity of Chinese CCEI

When conducting exploratory factor analysis, the cumulative variance contribution rate of the common factors needs to be greater than 60%, and the factor load of each item needs to be higher than 0.4 on its common factor, which suggests that each item can reflect the information of a certain dimension. The factor load capacity of each item and its dimensions in this study all exceeded 0.4. Among them, item 19 was swapped from FFA to PHO and item 20 from PHO to FFA. From the perspective of content relevance, item i.e. *Do you sometimes feel really panicky?*, is more appropriate in PHO dimension, whereas item 20 i.e. *Do you feel uneasy travelling on buses or the underground even if they are not crowded?*, falls more preferably into EFA. Community results of each dimension and the CCEI all exceeded 0.04, suggesting the components are explained well.

The CFA results show that almost every item had a load of above 0.4 under the category of its own factor, except for item 46 i.e. *Has your sexual interest altered?*. The factor load capacity of this item was 0.39, which may be caused by the fact that Chinese people have always been reserved about problems concerning sex, and the result of the survey may therefore be affected. However, considering the factor loading of item 46 is greater than 0.4 and the absolute fitting index was selected for the item to do confirmatory factor analysis, the fitting of the model is acceptable (Table 6), and the structure is ideal. The average variance extracted (AVE) of the combined reliability is preferably greater than 0.5, but if the residual variances (CR) of each dimension is greater than 0.6, an AVE greater than 0.4 is acceptable [27]. The CR values in this study are all greater than 0.6 and the AVE is greater than 0.4. And correlation coefficients among factors are all less than AVE, indicating that the items in the CCEI scale have a certain degree of discrimination and can identify the degree of response of different investigators (Table 7). It should be noted that this might be the first time that CFA is used in the evaluation of the structure of the CCEI questionnaire.

Through EFA and CFA, we finally determined that the Chinese version of CCEI contains six dimensions and a total of 47 items. The dimensions include depressive symptoms (items 5, 11, 17, 23, 29, 35, 41, 47), free-floating anxiety (items 1, 7, 13, 20, 25, 31, 37, 43), phobic anxiety (items 2, 8, 14, 19, 26, 32, 38, 44), somatic symptoms (items 4, 10, 16, 22, 28, 34, 40, 46), hysterical traits and symptoms (items 6, 12, 18, 24, 30, 36, 42, 48) and obsessive–compulsive traits and symptoms (items 3, 15, 21, 27, 33, 39, 45). Table 8 shows the reliability index of the Chinese CCEI is higher than that of the questionnaire in the original study by Crown and Crisp in 1966 [10]. This may be caused by the significant increase in the sample size of this study in comparison with that in the original study. Test–retest reliability is one of the most important methods to test the reliability of the CCEI questionnaire. The reliability result of the second survey 15 days after the first one, the reliability result from grouping odd and even numbered items, and the Cronbach’s αcoefficient are all greater than 0.7, indicating that the questionnaire has good internal consistency [28]. Considering the limitations of Cronbach’s α coefficient proposed by some researchers [29, 30], this study used ω coefficient, taking into consideration Omega coefficient, and the result was also greater than 0.7, which again shows the reliability of the internal consistency of the questionnaire.

Content validity is usually hinged upon experts’ review as the indication to the accuracy of the measured content or topic [31]. Typically, the validity is based on experts’ comments. I-CVI is the number of the experts scoring 4 or 5 for the importance of the research/total number of the experts. S-CVI is the number of items with a 4 or 5 score/total number of items [32]. In this research, the I-CVI and S-CVI all exceed 0.7, which suggests the Chinese CCEI has sound content validity. The reliability and validity indicators of this study show that the content and structure of the Chinese CCEI are fairly good, providing more options for clinical evaluation of patients’ psychology.

### CWP patients’ phobic anxiety

CWP patients generally have at least 20 years of exposure to dust at the time of diagnosis, and many are of retirement age; Chinese miners retire at 55 years old. It is worth noting that only the PHO dimension shows statistical significance. It is precisely because the patients are gradually accepting the fact that pneumoconiosis is a chronicle disease that cannot be cured and becoming accustomed to the decline in bio-activity caused by this disease. In fact, the miners are generally financially disadvantaged otherwise they would not have suffered from darkness, wet, dust, noise, high temperature and isolation in hundreds-deep mining pits. According to national policies [33], once pneumoconiosis is diagnosed, patients can enjoy free medical care, which in large part clears up patients’ worries. The hospital is like a free nursery where the patients can relax and have fun, such as playing chess and cards, jogging, watching TV, listening to audio books, and so on, although they have to use daily oxygen inhalation and alveolar washing in severer cases. These can explain why there is no significant difference in FFA, OBS, SOM, DEP, HYS among CWP patients and healthy retired miners. According to the survey results, the psychological difference between patients with pneumoconiosis and retired miners is reflected in PHO because of the fear produced by hypoxia, a respiratory impairment. For those who have experienced the torment of pneumoconiosis, they worry that they may not get help in time, especially when they are living alone. To improve patients’ psychological well-being and their lives, more accurate measurement tools and methods should be introduced to propose tailored psychological interventions.

## Conclusion

This study has demonstrated that the Chinese version of CCEI is of sound internal consistency and good structural validity and reliability. Though it was based on pneumoconiosis patients in this study, it is expected the Chinese version of CCEI can be applied to other types of patients to quickly assess psychological issues. It should be noted that due to occupational characteristics, for example, the subjects of this study are all male and generally old and from same places, in this study can be further improved by considering the variety of gender, age, education level, family income and location in the samples.

## Data Availability

All relevant data are reported within the paper. Analyzed data are available from the corresponding author on reasonable request.
